# Weighted Gene Coexpression Network Analysis in Mouse Livers following Ischemia-Reperfusion and Extensive Hepatectomy

**DOI:** 10.1155/2021/3897715

**Published:** 2021-12-30

**Authors:** Wei Liu, Yongquan Shi, Tao Cheng, Ruixue Jia, Ming-Zhong Sun, Shuqing Liu, Qinlong Liu

**Affiliations:** ^1^Department of Hepatobiliary Surgery, The Second Affiliated Hospital & College of Basic Medical Sciences, Dalian Medical University, Dalian, Liaoning, China; ^2^Department of Traditional Chinese Medicine, The First Affiliated Hospital of Dalian Medical University, Dalian, China; ^3^Department of Pathology, Jinan Central Hospital Affiliated to Shandong University, Jinan, Shandong, China

## Abstract

In mouse models, the recovery of liver volume is mainly mediated by the proliferation of hepatocytes after partial hepatectomy that is commonly accompanied with ischemia-reperfusion. The identification of differently expressed genes in liver following partial hepatectomy benefits the better understanding of the molecular mechanisms during liver regeneration (LR) with appliable clinical significance. Briefly, studying different gene expression patterns in liver tissues collected from the mice group that survived through extensive hepatectomy will be of huge critical importance in LR than those collected from the mice group that survived through appropriate hepatectomy. In this study, we performed the weighted gene coexpression network analysis (WGCNA) to address the central candidate genes and to construct the free-scale gene coexpression networks using the identified dynamic different expressive genes in liver specimens from the mice with 85% hepatectomy (20% for seven-day survial rate) and 50% hepatectomy (100% for seven-day survial rate under ischemia-reperfusion condition compared with the sham group control mice). The WGCNA combined with Kyoto Encyclopedia of Genes and Genomes (KEGG) and Gene Ontology (GO) enrichment analyses pinpointed out the apparent distinguished importance of three gene expression modules: the blue module for apoptotic process, the turquoise module for lipid metabolism, and the green module for fatty acid metabolic process in LR following extensive hepatectomy. WGCNA analysis and protein-protein interaction (PPI) network construction highlighted FAM175B, OGT, and PDE3B were the potential three hub genes in the previously mentioned three modules. This work may help to provide new clues to the future fundamental study and treatment strategy for LR following liver injury and hepatectomy.

## 1. Introduction

Partial hepatectomy is a routine surgical procedure in liver resection, trauma, and transplantation. Liver ischemia-reperfusion injury commonly happens as one of the main causes of acute liver dysfunction and failure after partial hepatectomy and liver transplantation [1, 2]. However, the precise mechanisms underlying ischemia-reperfusion injury have been poorly elucidated. Liver function cannot be fully restored after ischemia-reperfusion injury [3]. Partial hepatectomy and ischemia-reperfusion trigger hypovolemic shock in liver as an inevitable complication and a significant challenge for clinical management [4]. The investigation on the gene expression alterations with the underlying molecular mechanisms in ischemia-reperfusion injury helps us to manage and control iatrogenic liver damage and dysfunction. Commonly, partial (two-thirds) hepatectomy is used to resect diseased livers [5], while a larger proportion of liver resection is required for liver donation. Moreover, the progress on liver regeneration (LR) already made the successful treatments of liver cancer and liver cirrhosis with extensive hepatectomy and the feasibility of liver transplantation [6]. In this study, we used mouse models with 50% or 85% hepatectomy accompanied with ischemia-reperfusion to identify the differentially expressed genes (DEGs) in liver tissues. For liver resection, the classic regeneration model is the 2/3 liver resection model established in 1931 by Higgins and Anderson. We simulated the surgical method of clinical liver resection by combining hepatectomy under ischemia-reperfusion. We achieved the seven-day survival rates of 100% and 20% for the mice treated with 50% and 85% hepatectomy under ischemia-reperfusion condition, respectively. Meanwhile, the sham group mice were also included as control for experiment group mice. Comparisons between the sham group and 50% and 85% hepatectomy reflected the dynamic gene expression changes in LR. Herein, we explored an 85% of hepatectomy in mice and aimed to investigate the molecular mechanisms and provide a theoretical basis to expand surgical hepatectomy ratio. The weighted gene coexpression network analysis (WGCNA), a highly effective method for the construction of coexpression networks by using large-scale datasets and for the rapid identification of genes with close associations in functions [7], was performed in current work. The relevant gene modules and hub genes in important biological processes were also extensively explored using WGCNA in the combination with KEGG and GO enrichment analyses related to liver regeneration. The DEGs together with their potential interaction mechanisms raveled in current work provide new clues to the future fundamental study and treatment strategy for LR following liver injury and hepatectomy.

## 2. Materials and Methods

### 2.1. Animals and Experiments

The animal protocol was approved by the Institutional Animal Care and Use Committee (IACUC) of Dalian Medical University (Dalian, China) and was carried out in accordance with the Guidelines of the Care and Use of Laboratory Animals issued by the Chinese Council on Animal Research (Approval Number: AEE19042 2019). Male C57BL/6J mice (8–10 weeks of age, ～19 g) were obtained from the specific pathogen-free (SPF) Animal Laboratory Center of Dalian Medical University (Dalian, China). The experiments were divided into seven groups with three mice in each group. The seven groups were sham group, sham operation, 0 h; test 1 group, ischemia-reperfusion 50% hepatectomy at 6 h; test 2 group, ischemia–reperfusion 50% hepatectomy at 12 h; test 3 group, ischemia-reperfusion 50% hepatectomy at 24 h; test 4 group, ischemia-reperfusion 85% hepatectomy at 6 h; test 5 group, ischemia-reperfusion 85% hepatectomy at 12 h; test 6 group, ischemia-reperfusion 85% hepatectomy at 24 h (Table 1). In the sham group, only the mouse abdominal cavity was opened, the liver lobes were turned without resection, and samples were taken at 0 h. Mice were anesthetized by inhalation with Sevoflurane (4.0% v/v; Maruishi Pharmaceutical, Japan), maintained on 37°C thermostatic heat pads, and underwent laparotomy. The hepatic portal veins were occluded with a vascular clamp for 20 min and hepatectomy was performed at the same time. Based on our preliminary but extensive experimental trials, we ensured that the longest time required for 85% hepatectomy was 20 min. In order to eliminate the effect of clamping time on mouse, we uniformed the clamping time of 20 min for each mouse. The skin and connective tissue beneath and around the xiphoid process were dissected in prior to open the abdominal cavity, and two parallel incisions of the left and right xiphoid process were made to expose the liver. The surgeon used sterile 5–0 silk ligatures to isolate both lobes simultaneously as close as possible to the inferior vena cava (IVC), dissected the tissue distal to the ligature, and occluded the hepatic portal vein and hepatic artery with a vascular clamp for 20 min during hepatectomy. 50% hepatectomy included the resections of the left and the half of the middle liver lobe, while 85% hepatectomy included 50% hepatectomy plus all the middle, right lower, and caudate liver lobes [8]. The mice were allowed to recover for 30 min before being sacrificed by cervical transection. Tissues were harvested at the designated time point and stored or processed accordingly (see Table 1 for more details). Every effort was deployed to minimize pain.

### 2.2. RNA Isolation and mRNA Microarray Analysis

Snap-frozen liver tissues were ground and subjected to total RNA isolation using Trizol reagent (Invitrogen, Carlsbad, CA, USA), purified with a RNeasy mini kit from Qiagen (Valencia, CA, USA), and quantified with a spectrophotometer and bioanalyzer 2000 (Agilent Technologies, Santa Clara, CA). The RNA samples (150 ng each) were subjected to reverse transcription to generate biotinylated cDNA probes using the Ambion® WT Expression Kit (Austin, TX, USA). The fragmented cDNA probes (denatured at 95°C for 3 min) were hybridized at 45°C for 16 h onto Gene Chip Affymetrix Mouse Clariom® D Arrays (Affymetrix, Santa Clara, CA) in accordance with the manufacturer's protocol. The following day, the gene chips were washed and stained in the Affymetrix Fluidics Station 450, in accordance with the standard Affymetrix protocol. The gene chips were scanned using an Affymetrix® Gene Chip® Scanner 3000 7G with the Gene Chip Command Console (AGCC), and the data were analyzed using the robust multichip analysis (RMA) algorithm with default Affymetrix settings and global scaling for normalization. One-way ANOVA test was used to identify DEGs among the seven groups of samples. The thresholds for upregulation and downregulation of DEGs were set as a fold change >1.5 and a *P* value < 0.05. We identified 5,312 differentially expressed genes (DEG). These microarray data are publicly available at NCBI Gene Expression Omnibus (GEO) with the accession number GSE133271.

### 2.3. Function Enrichment Analyses

GO (https://geneontology.org/) and KEGG pathway (https://www.kegg.jp/) analyses were performed for DEGs. The results from three gene modules in apoptotic process, in lipid metabolism, and in fatty acid metabolic process were obtained and visualized by the R package software (ggplot version 2 3.10). Statistical analysis involved Fisher's exact test (*P* < 0.05), and the Benjamini–Hochberg method was used to correct FDR < 0.05.

### 2.4. Weighted Gene Coexpression Network Construction

WGCNA is an algorithm based on high-throughput gene expression profiling [9, 10]. It is most suitable for gene coexpression network analysis for revealing the correlation between genes and related gene function modules. In this study, we used WGCNA in the R environment [11] to construct a scale-free coexpression network of DEGs in mice livers after receiving different proportions of liver resection plus ischemia-reperfusion. The Data Analysis Hierarchical Clustering Tab is a powerful and useful tool that can be used to analyze large genomic datasets and evaluate three types of DEGs: binary, aggregate, and hierarchical clustering. The first step of hierarchical clustering is to calculate the distance matrix between gene expression data. Aggregation level processing consists of repeated loops, where the two closest remaining items (items with the smallest distance) are connected by nodes/branches of the tree, and the length of the branches determines the distance between the connected items. After that, the two merged projects are deleted from the list of projects being processed and replaced with projects that represent the new branch. Then, we calculated the distance between the new item and all other remaining items and repeated the process until only one item remained. We set the soft threshold power *β* as 21 and the scale-free *R*^2^ as 0.90 to construct a standard scale-free network with the function of “soft threshold.”

### 2.5. Identifying Significant Modules and Identification of Candidate Hub Genes

First, we calculated the correlation between ME and each model group to identify related modules. Modular intrinsic genes (MEs) were defined as the first major component of the gene expression matrix of each corresponding module. We focused on the three most relevant modules for test 4, test 5, and test 6 to be shown in blue, turquoise, and green (*P* < 0.05). In the linear regression between gene expression and the experiment group, the gene significance (GS) was defined as log_10_*P* (GS = lgP). We defined the module signal (MS) as the average of the GSs of all genes in the module [12]. We also measured the absolute value of Pearson's correlation (|MM| > 0.8) to indicate gene connectivity. Module membership (MM) was defined as the correlation between module characteristic genes (ME) and gene expression. Central genes highly correlated with time were then identified in the module with the absolute value of Pearson's correlation (|GS|) over 0.2. Therefore, based on |GS| > 0.2 and |MM| > 0.8, we were able to identify hub genes.

### 2.6. Coexpression of mRNA and mRNA

The correlation coefficient of mRNA-mRNA pairs was used to construct a coexpression network [13]. The centrality degree of each gene was calculated by Cytoscape 3.6.0 neutral analyzer (https://cytoscape.org/). Then, we selected top ten genes according to the centrality degree as our hub genes [14] for further analysis.

### 2.7. Statistical Analysis

All the experimental data are summarized as mean ± standard deviation (SD) and were analyzed using Student's *t*-test andone-way ANOVA test. *P* < 0.05 was considered statistically significant.

## 3. Results

### 3.1. Identification of Differentially Expressed Genes (DEGs)

**Figure 1:**
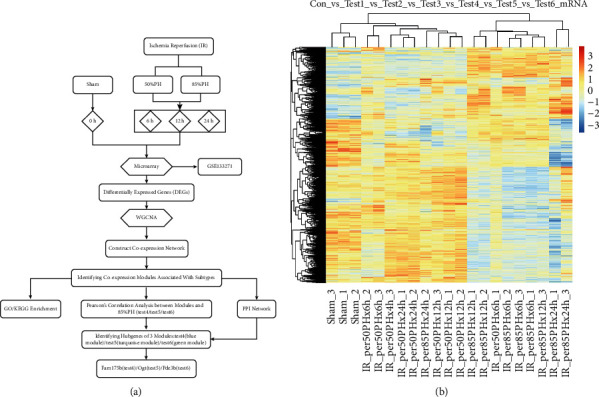
Study design and hierarchical cluster analysis. (a) Analysis procedure flowchart: model establishment, data collection, preprocessing, analysis, and verification. (b) Hierarchical cluster analysis of 5,312 DEGs in mice in the sham, test 1, test 2, test 3, test 4, test 5, and test 6 groups.

The workflow of our study is shown in Figure 1(a). In the model of ischemia-reperfusion hepatectomy (sham operation vs. test 1 vs. test 2 vs. test 3 vs. test 4 vs. test 5 vs. test 6), we screened 5,312 DEGs under the threshold of |FC| > 1.5 (*P* < 0.05; Figure 1(b)). These DEGs were then used for subsequent analysis.

### 3.2. Weighted Coexpression Network Construction and Identification of Key Modules

**Figure 2:**
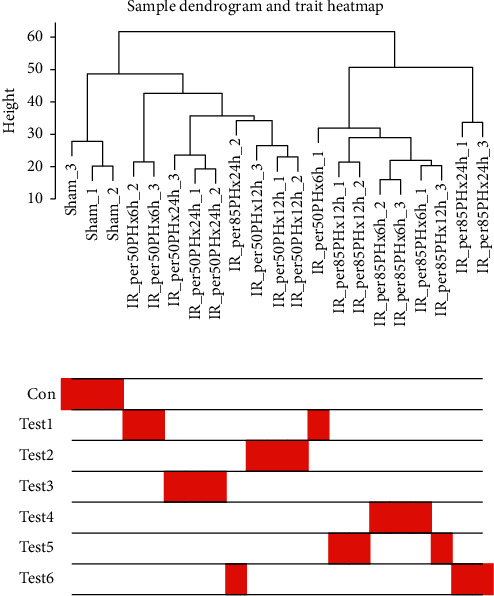
Sample dendrogram and trait heatmap.

**Figure 3:**
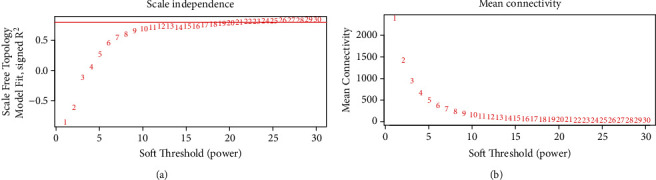
Determination of the soft-thresholding power (*β*) in WGCNA. (a) Analysis of the scale-free topology model fitting index (*R*^2^, *y*-axis). (b) Mean connectivity for various soft-thresholding powers. The red Arabic numbers denote different soft thresholds. There was a trade-off between maximizing *R*^2^ and maintaining a high mean number of connections. *β* was set as 21.

**Figure 4:**
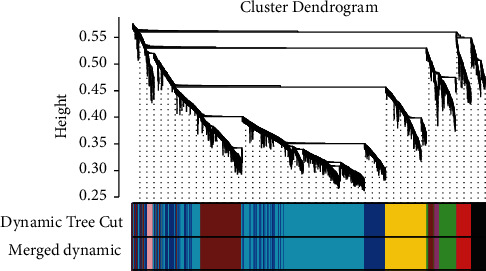
Dendrogram of the clustering of all DEGs based on dissimilarity measures.

**Figure 5:**
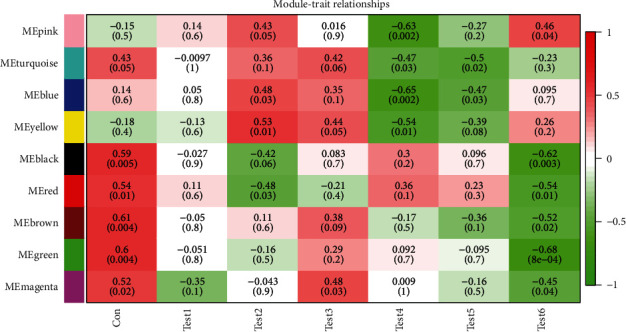
Heat map shows the correlation between characteristic genes in the liver resection module and each test group. Each column shows the corresponding correlation and *P*-value.

Clustering of the DEGs was based on sham, test 1, test 2, test 3, test 4, test 5 and test 6 groups as separately marked with color bars in Figure 2. Before the construction of the weighted coexpression network, a weighted parameter of the adjacency function (soft-threshold *β* = 21) was selected to build the scale-free networks as shown in Figures 3(a) and 3(b). The data demonstrated that the closer was for the branches, and the more similar was for their expression profiles. Nine coexpressed gene modules were detected by dynamic tree cutting and merging similar modules, which were visualized by a clustering dendrogram (Figure 4). We found that all nine modules and sample types showed clear correlations (Figure 5). The brown, yellow, magenta, blue, turquoise, and green modules were found most relevant to the sham, test 2, test 3, test 4, test 5 and test 6 groups, respectively. As the results showed that the last three modules were negatively correlated with DEGs in test 4, 5, and 6 groups, later on, we mainly focused and explored the significances of these modules in mouse LR receiving the extensive liver resection of 85%.

**Figure 6:**
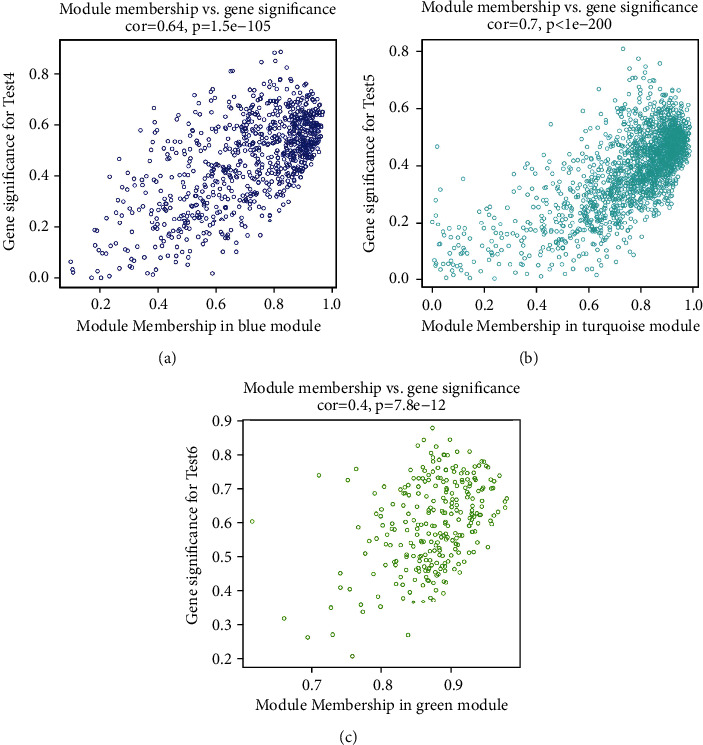
Dot scattering plots of highly correlated modules at different time points in the 85% partial resection mice. (a) The blue module was correlated with test 4; (b) the turquoise module was correlated with test 5; (c) the green module was correlated with test 6.

Based on the combinational analyses of module connectivity and the absolute value of Pearson's correlation, we identified that the blue module, turquoise module, and green module showed the highest level of correlation for test 4, test 5, and test 6, respectively (Figures 6(a)–6(c)). The blue, turquoise, and green modules were the most meaningful modules specifically in the apoptotic, lipid metabolism, and fatty acid metabolic processes, which were demonstrated to be closely related to mouse LR after an 85% hepatectomy for 6, 12, and 24 h, respectively. These modules were then used to identify the hub genes.

### 3.3. Functional Enrichment Analysis of the Hub Genes in WGCNA

**Figure 7:**
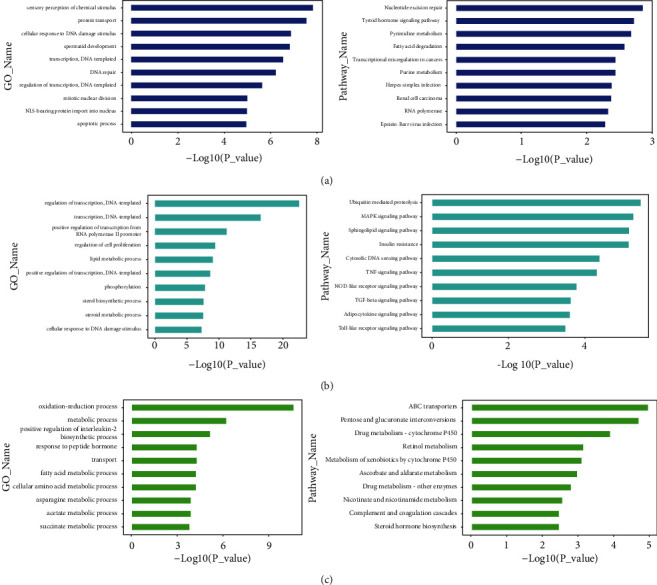
Analysis of the top ten GO (BP biological process) and KEGG pathways for each module. (a) Blue module, (b) turquoise module, and (c) green module. The *x*-axis indicates the –log 10 (*P*-value), while the *y*-axis indicates the GO or KEGG pathway. GO: gene ontology; KEGG: Kyoto Encyclopedia of Genes and Genomes.

We performed GO and KEGG pathway analyses on the DEGs identified in the 3 modules. The top ten blue modules for biological processes and pathways, as determined by GO enrichment analysis, are shown in Figure 7(a), including sensory perception of chemical stimulus, protein transport, cellular response to DNA damage stimulus, spermatid development transcription, DNA templating, DNA repair, regulation of transcription, mitotic nuclear division, NLS-bearing protein import into nucleus, and apoptotic process. The top ten KEGG pathways included the cell cycle, nucleotide excision repair, thyroid hormone signaling pathways, pyrimidine metabolism, fatty acid degradation, purine metabolism, transcriptional misregulation in cancer, herpes simplex infection, renal cell carcinoma, RNA polymerase, and Epstein-Barr virus infection. Apoptosis is a form of programmed cell death that occurs in both physiological and disease states. Under normal conditions, apoptosis and cell proliferation are complementary and necessary for cell maintenance, growth, and degradation. The regulation of apoptosis is critical in LR process [15, 16]. Damage to LR in response to aging is accomplished by reducing cell cycle activity and by increasing the levels of autophagy and apoptosis [17]. MMP-9 plays an important role in liver regeneration after partial hepatectomy; it is possible that this protein regulates changes in pathways related to proliferation and apoptosis [18].

The top biological processes (BPs) arising from GO enrichment and KEGG pathway analyses in the turquoise module are shown in Figure 7(b), including lipid metabolism, sterol biosynthetic processes, steroid metabolic processes and insulin resistance, and the adipocytokine signaling pathway. The top 10 BPs arising from GO enrichment and KEGG pathway analysis in the green module are shown in Figure 7(c). Among them, the fatty acid metabolic process, pentose and glucuronate interconversions, and steroid hormone biosynthesis involved in mouse LR following 85% hepatectomy in 24 h. According to our analyses, the functions and signal pathways in the metabolism of sugar and fat played crucial roles in test 5 and test 6. The regenerated liver quickly acquires the ability to mobilize triglycerides as the larger normal liver to process free fatty acids [19]. Fatty tissue lipids are liver neutral lipids formed during LR and represent the source of fatty acids contained in phospholipids [20]. Prior to the peak period of proliferation, the regenerating liver undergoes a characteristic period of transient lipid accumulation in hepatocytes. Our current results showed that 24 h after 85% hepatectomy, the storage of lean and adipose tissues both decreased dramatically, while only little was changed for these factors in the control mice. At 24 h after surgery, the quality of lean tissue had decreased by 10% and the mass of fat had decreased by 20%. These catabolic changes were previously reported to occur after the onset of hypoglycemia, approximately three hours after partial hepatectomy [21]. Transient regeneration-associated steatosis (TRAS) was reported as an indispensable aspect of successful LR, in which fat acted as the main fuel for regeneration during the periods of low liver function. The increase in lipid catabolism during LR is a promising option for clinical management [22]. The ability of mice to undergo complete LR after partial hepatectomy is known to depend on the ability of liver cells to acquire glucose [23].

### 3.4. Hub Gene Identification through WGCNA and PPI

**Figure 8:**
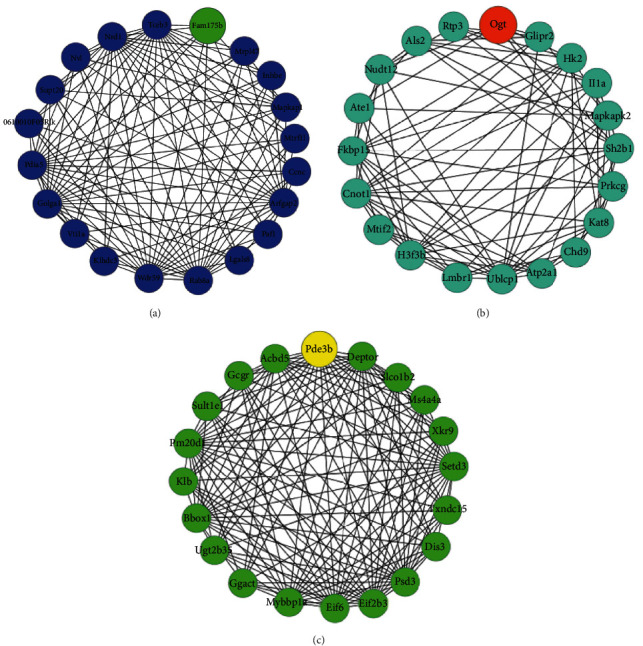
Network analyses of the blue (a), turquoise (b), and green (c) modules. Only the top 20 hub genes were displayed. All gene network diagrams are available in the Supplementary Files (available here).

**Figure 9:**
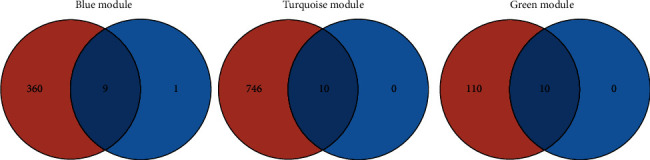
Common hub genes in the coexpression and PPI networks. A Venn diagram was utilized to screen the hub genes between the DEGs and WGCNA. Twenty-nine genes were screened as candidates for further analysis and validation.

Cytoscape software established a coexpression network to show the common genes among the modules (Figure 8). We analyzed the blue, turquoise, and green modules to get the top ten genes for each module according to the centrality degree value. 360, 746, and 110 hub genes in the coexpression network were obtained for the blue, turquoise, and green modules, respectively, as shown in Figure 9. A total of 29 genes, including Arfgap2, Golga1, Tceb3, Pdia5, Wdr59, Rab8a, Mapkap1, Nrd1, Fam175b, Mapkapk2, Kat8, Rt-p3, Mtif2, Fkbp15, OGT, Als2, H3f3b, Ublcp1, Cnot1, Eif6, Bbox1, Setd3, Mybbp1a, Pde3b, Eif2b3, Psd3, Acbd5, Deptor and Pm20d1, were highlighted by both WGCNA and PPI network analyses. Then, the overlapping regions among the three modules by using BP functional enrichment and KEGG pathways were used to pick up specific target genes of interest. In the blue module, we found that Fam175b gene showed BP enrichment and acted as a negative regulator of apoptosis. At 6 h, Fam175b was analyzed with a most expression reduction. In the turquoise module, BP GO enrichment and KEGG pathway enrichment assays provided OGT as a hub gene. It showed an utmost expression decrease at 12 h. The main function of OGT is to regulate the insulin receptor signal transduction pathway, gluconeogenesis in cell glucose homeostasis, response to insulin, and protein O-linked glycosylation. In the green module, the hub gene was selected as Pde3b with the largest decrease degree in expression at 24 h. Pde3b plays key roles in angiogenesis, glucose homeostasis, cellular response to insulin stimulus, insulin secretion, cell adhesion, CAMP catabolic process, lipid catabolic process, endocrine pancreas development, and signal transduction. We focused particularly on its negative regulation of lipid catabolic processes.

## 4. Discussion

Globally, liver cancer ranks as one of most common causes of death. 85,000 new cases of liver cancer increase yearly worldwide. Surgical operations for the treatment of liver diseases including liver cancers often require major hepatectomy. Liver insufficiency, together with especially the liver failure after the hepatectomy surgery, is a major challenge for liver surgeons. One of the most significant issues is that we do not know how the liver regenerates following liver resection, particularly regarding the specific action mechanisms. Mouse models of liver regeneration (LR) have been extensively reported and most of the studies predominantly focused on LR and related gene alterations [24–26], while the mouse models did not fully consider or mimic clinical issue of liver damage and dysfunction induced by ischemia-reperfusion used in liver surgical operation. The appropriate mouse model with different expressive genes in their livers collectable through surviving extensive hepatectomy with ischemia-reperfusion will be of critical importance in LR for treating various liver diseases.

In current study, we designed an ischemia-reperfusion hepatectomy mouse model of 85% hepatectomy with the survival rate of ∼20%. Using Affymetrix gene-chip assays, we identified over 5000 DEGs among the seven different experiment groups. Encouragingly, the combinational analyses using WGCNA, KEGG, and GO platforms pinpointed out three gene expression modules: the blue module for apoptotic process, the turquoise module for lipid metabolism and the green module for fatty acid metabolic process in mouse liver following 85% extensive hepatectomy at the time intervals of 6, 12 and 24 h, respectively.

Apoptosis plays a central role in regulating liver development and homeostasis. It can reflect the severity of liver injury. The degree of liver cell apoptosis is an important parameter of liver quality [27]. Apoptosis is an early, chronic, and temperate response that occurs following the initiation of injury. The interference on the apoptosis of liver cells can delay disease progression and reduce liver dysfunction. However, there is currently no such treatment available in clinical practice. To treat premature cell death, it may be possible to consider the key components that inhibit apoptosis, such as caspase [28]. FAM175 family was reported to negatively regulate the apoptotic process. The activation of FAM175 in the early stages after hepatectomy could inhibit cell apoptosis and reduce liver damage, thereby increasing LR and improving survival rate. FAM175B, also known as ABRO1/KIAA0157, is a member of the FAM175 family and was originally reported as a component of the BRCC36-containing isopeptidase complex (BRISC) deubiquitinating enzyme complex. In this complex, FAM175B acts as a scaffold protein for BRISC to recruit proteins and promote its deubiquitination activity. FAM175B acts as a tumor suppressor in esophageal squamous cell carcinomas (ESCC). The action of this protein is mediated by inhibiting the ubiquitin-dependent degradation of ATF4 and promoting apoptosis [29]. FAM175B regulates the stability of p53 protein and increases the latter's transcriptional activity in apoptosis. It cooperates with p53 in the mediation of apoptosis [30]. Under normal conditions, the basal level of apoptosis in cells transfected with GFP-vector was 12.5% versus 4% in cells transfected with GFP-FAM175. When the cells were treated with H_2_O_2_, 71% of the cells transfected with GFP-vector were apoptotic compared with 44% of the cells transfected with GFP-FAM175. Consequently and interestingly, the overexpression of FAM175B protects cells from apoptosis induced by oxidative stress [31, 32]. Herein, current work shows that FAM175B, acting as a critical hub gene, is involved in mouse LR with extensive hepatectomy through acting on apoptosis process. Its downregulation after hepatectomy potentially improves the growth of liver cells via inhibiting their apoptosis in LR. It is of importance in the fields of liver injury treatment, liver partial resection, and liver transplantation.

LR requires the initial synthesis of large amounts of lipids [33]. Some experiments have reported the downregulation of lipid-related genomic pathways during the early stages of LR [34]. Our work showed that the turquoise module for lipid metabolism was emphasized in mouse liver following 85% extensive hepatectomy at 12 h with OCT as a core hub gene. OGT was first isolated in 1992 from rat liver [35] and is best known for its role in glycosylating nuclear and cytoplasmic proteins. OGT is necessary for cell viability [36–39]. In the liver and skeletal muscle of OGT MKO mice, the levels of TAG increased by a factor of 2–3 fold in the mice's fatty livers with heavier liver weight, lighter liver color, and steatosis [40]. Interestingly, compared with control mice, the plasma TAG and cholesterol levels were increased in mice that overexpressed OGT. It was reported that OGT suppressed Insig-1, a negative regulator of lipid synthesis, and induced insulin resistance and dyslipidemia via PIP-dependent insulin signal disturbances [41]. Our work shows OGT is downregulated in the liver of mice after 85% hepatectomy. It is clear that acting as a core hub gene, OGT is involved in mouse LR through mediating lipid metabolism process. It provides a new clue to the study on liver injury, partial resection, and transplantation.

Current work revealed the involvement of fatty acid metabolic process (the green gene module) in mouse LR after 85% extensive hepatectomy for 24 h. Cyclic nucleotide phosphodiesterase (PDE) is an important regulator of the signal transduction process mediated by cAMP and CGMP. PDEs belong to a complex and diverse superfamily containing at least 11 structurally related, highly regulated, and functional different genes (PDE1–PDE11) [42]. PDE3 is encoded by two genes (PDE3A and PDE3B). It plays an important role in the energy metabolism of different types of cells including hepatocytes, brown and white adipocytes, and pancreatic *β* cells [43, 44]. PDE3B plays important roles in triglyceride and cholesterol biosynthesis in the liver fat cells and liver fat formation [45–47]. In obese insulin resistant db/db mouse, PDE3B was increased in mouse liver but decreased in adipose tissue [45]. In PDE3B-KO mice, the triglyceride (TG) level was significantly higher in mice livers with increased expression of fatty acid synthase (FAS) [46]. Herein, our work establishes the association of PDE3B with LR through fatty acid metabolic process. It is important in the research and treatment of liver diseases.

## 5. Conclusions

In this study, we used the ischemia-reperfusion 85% hepatectomy mouse model to screen the differentially expressed genes (DEGs) in livers and used the bioinformatic assessment to address molecular events in liver regeneration (LR) after extensive hepatectomy. Three key gene expression modules were used: the blue module for apoptotic process, the turquoise module for lipid metabolism, and the green module for fatty acid metabolic process in mouse LR following an extensive 85% hepatectomy for 6, 12, and 24 h, respectively. WGCNA analysis and protein-protein interaction (PPI) network construction highlighted that FAM175B, OGT, and PDE3B were the potential core hub genes separately for the blue, turquoise, and green modules. This work provides new clues to the future fundamental study and treatment strategy for liver injury, partial resection, and transplantation.

## Figures and Tables

**Table 1:** Experimental grouping for mouse partial hepatectomy.

Sample	Partial hepatectomy	Time

Sham	Sham operation	0 h
Test 1	50% hepatectomy with ischemia-reperfusion 20 min	6 h
Test 2	50% hepatectomy with ischemia-reperfusion 20 min	12 h
Test 3	50% hepatectomy with ischemia-reperfusion 20 min	24 h
Test 4	85% hepatectomy with ischemia-reperfusion 20 min	6 h
Test 5	85% hepatectomy with ischemia-reperfusion 20 min	12 h
Test 6	85% hepatectomy with ischemia-reperfusion 20 min	24 h

## Data Availability

The raw data and GEO files used to support the findings of this study are available in the Figshare Repository at the following links: https://figshare.com/s/117f9001ee0e1cd7eda8. https://figshare.com/s/4dbff17f468d2bdbf935. These microarray data are publicly available at NCBI Gene Expression Omnibus (GEO) with the accession number GSE133271.

## Supplementary Materials

Supplementary Materials Network analysis shows the central gene network of the blue module, turquoise module, and green module. The node represents the gene, and the node size represents the moderate score. The edges are colored by weight: (A) blue module; (B) turquoise module; (C) green module.
