# Supplementary material for: Weighted Gene Coexpression Network Analysis in Mouse Livers following Ischemia-Reperfusion and Extensive Hepatectomy
Source: Evid Based Complement Alternat Med. 2021 Dec 30;2021:3897715. doi: 10.1155/2021/3897715 (PMC8736699; doi:10.1155/2021/3897715)
Supplement: Supplementary Materials — Network analysis shows the central gene network of the blue module, turquoise module, and green module. The node represents the gene, and the node size represents the moderate score. The edges are colored by weight: (A) blue module; (B) turquoise module; (C) green module. [file 3897715.f1.pdf]

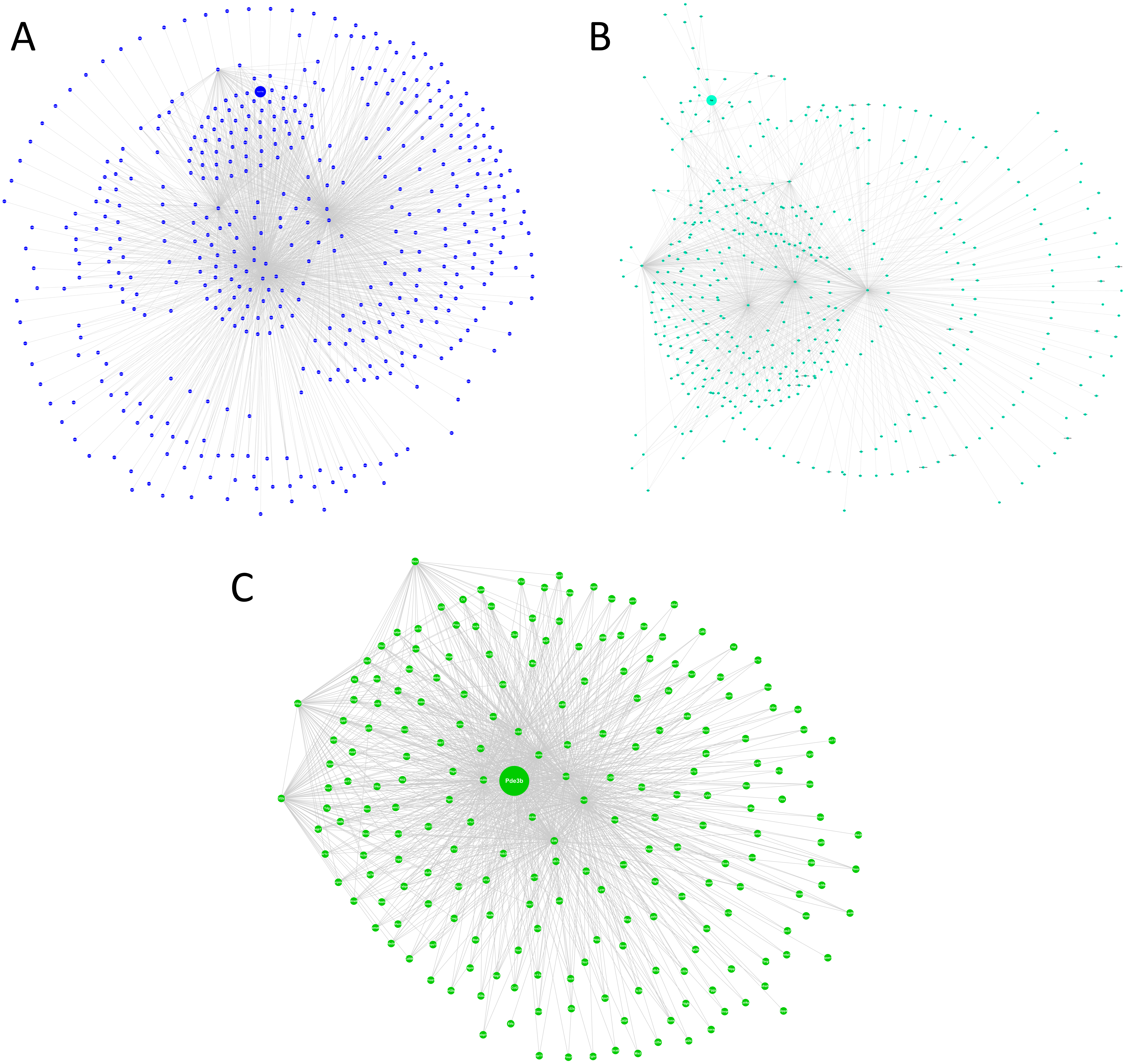
A
B
C
Pde3b
